# Protein Tyrosine Phosphatase-1B Modulates Pancreatic β-cell Mass

**DOI:** 10.1371/journal.pone.0090344

**Published:** 2014-02-28

**Authors:** Rebeca Fernandez-Ruiz, Elaine Vieira, Pablo M. Garcia-Roves, Ramon Gomis

**Affiliations:** 1 Diabetes and Obesity Research Laboratory, Institut d’Investigacions Biomediques August Pi i Sunyer (IDIBAPS), Barcelona, Spain; 2 Centro de Investigación Biomédica en Red de Diabetes y Enfermedades Metabólicas Asociadas (CIBERDEM), Barcelona, Spain; 3 Universitat de Barcelona, Barcelona, Spain; 4 Hospital Clinic de Barcelona, Barcelona, Spain; St. Vincent's Institute, Australia

## Abstract

Protein tyrosine phosphatase 1B (PTP1B) is a negative regulator of the insulin signalling pathway. It has been demonstrated that PTP1B deletion protects against the development of obesity and Type 2 Diabetes, mainly through its action on peripheral tissues. However, little attention has been paid to the role of PTP1B in β-cells. Therefore, our aim was to study the role of PTP1B in pancreatic β-cells. Silencing of PTP1B expression in a pancreatic β-cell line (MIN6 cells) reveals the significance of this endoplasmic reticulum bound phosphatase in the regulation of cell proliferation and apoptosis. Furthermore, the ablation of PTP1B is able to regulate key proteins involved in the proliferation and/or apoptosis pathways, such as STAT3, AKT, ERK1/2 and p53 in isolated islets from PTP1B knockout (PTP1B ^−^/^−^) mice. Morphometric analysis of pancreatic islets from PTP1B ^−^/^−^ mice showed a higher β-cell area, concomitantly with higher β-cell proliferation and a lower β-cell apoptosis when compared to islets from their respective wild type (WT) littermates. At a functional level, isolated islets from 8 weeks old PTP1B ^−^/^−^ mice exhibit enhanced glucose-stimulated insulin secretion. Moreover, PTP1B ^−^/^−^ mice were able to partially reverse streptozotocin-induced β-cell loss. Together, our data highlight for the first time the involvement of PTP1B in β-cell physiology, reinforcing the potential of this phosphatase as a therapeutical target for the treatment of β-cell failure, a central aspect in the pathogenesis of Type 2 Diabetes.

## Introduction

Type 2 Diabetes is currently considered one of the main causes of morbidity and mortality worldwide. Insulin resistance is a key characteristic in the development of the disease, and it is defined as the diminished ability of cells to respond to insulin in terms of glucose uptake and utilization in peripheral tissues [1]. At the prediabetic stages, pancreatic β-cell is able to counteract insulin resistance by increasing insulin secretion in order to maintain normoglycaemia [2]. However, as the disease progresses, β-cell compensation fails and β-cell mass is reduced and glucose homeostasis is disrupted [3], [4].

The mechanism underlying the β-cell compensatory response in humans is still unclear. Evidences from animal studies suggest that the insulin signalling pathway could be critical for β-cell growth and survival [5], [6]. Insulin signalling is tightly regulated by the phosphorylation status of several components of the critical nodes and pathways that control its actions, being relevant both kinases and phosphatases enzymes [7]. One of the most important phosphatases regulating the insulin signalling cascade is the protein tyrosine phosphatase 1B (PTP1B) which inhibits insulin receptor and insulin receptor substrate 1 by direct dephosphorylation [8]. Previous studies performed in PTP1B whole body knockout mice (PTP1B ^−^/^−^) have revealed that these mice are hypersensitive to insulin and resistant to weight gain on a high-fat diet [9], [10]. Studies using PTP1B tissue-specific knockout mouse models defined key actions of this phosphatase to regulate whole body energy and glucose homeostasis in brain, adipose tissue, liver and muscle [11]-[14].

Although the beneficial effects of targeting PTP1B in peripheral tissues are evident the role of PTP1B in pancreatic β-cell is not fully understood and the information regarding this issue is scarce [15], [16]. In this study, we provide new insights into how PTP1B is able to regulate important signalling pathways involved in both β-cell proliferation and survival. We also give evidences about the role of PTP1B in regulating β-cell mass and function under physiological and pathological (streptozotocin-induced diabetes) conditions *in vivo*. Our results led us to propose that strategies aimed to antagonize the effects of PTP1B would be beneficial not only in peripheral tissues but also at the level of pancreatic β-cell.

## Materials and Methods

### Ethics Statement

All animal procedures were approved by the Animal Ethics/Research Committee of the University of Barcelona, and principles of laboratory animal care were followed.

### Cell culture studies

The insulin releasing MIN6 β-cell line was kindly provided by Dr. Jun-Ichi Miyazaki (Osaka University, Osaka, Japan), and used between passages 20 and 30 [17]. MIN6 β-cells were maintained in DMEM containing 25 mmol/l glucose, 10% FBS, 50 Units/ml Penicillin, 50µg/ml Streptomycin, 2 mM L-glutamine, and 50µM β-mercaptoethanol. PTP1B silencing was achieved by using a siGENOME SMART pool, mouse ptpn1 (Thermo Fisher Scientific, Inc, Waltham, MA, USA) and a scramble siRNA (Applied Biosystems, Life Technologies Ltd, Paisley PA4 9RF, UK) as a control. SMARTpool^TM^ siRNA pools four highly functional SMART selection designed siRNA targeting the same gene, minimizing off-target effects. Cells were transfected for 48 hours by using the transfection reagent Metafectene Pro (Biontex, Martinsried/Planegg, Germany). Transfected MIN6 β-cells were subsequently used for protein and RNA extraction, and for performing an *in vitro* cell proliferation assay.

### RNA isolation and quantitative PCR analysis

Total RNA was isolated from transfected MIN6 cells by using RNeasy Mini Kit (QIAGEN, Hilden, Germany) and quantified using a Nanodrop 1000 (Thermo Scientific, Wilmington, MA). RNA was reverse transcribed using High Capacity cDNA Reverse Transcription Kit (AB Applied Biosystems) following the manufactureŕs instructions. Real time PCR was carried out in Light Cycler 480 System ((Roche, Basel, Switzerland).) using MESA GREEN qPCR MasterMix Plus for SYBR Assay (Eurogentec, Liège, Belgium). mRNA expression levels were determined using the Standard Curve Method and normalized to the expression of acidic ribosomal protein 36B4 gene. Primer sequences are the following: ptpn1 forward: CATCCAGAACATGCAGAAGCCGCT and reverse: TTCCCAGCCTTGTCGATCTC; 36B4 forward: GAGGAATCAGATGAGGATATG and reverse: AAGCAGGCTGACTTGGTTGC. Gene expression in si-ptpn1 MIN6 cells was expressed relative to the mean of the values of the same gene found in scramble MIN6 cells, set at value 1 as the reference.

### In vitro cell proliferation assay

To assess *in vitro* cell proliferation in control (Sc) and si-ptpn1 MIN6 cells, we have performed a colorimetric assay based on the measurement of the incorporation of BrdU, an analogue to thymidine, during DNA synthesis in proliferating cells. We have followed manufacturer's instructions (Cell proliferation ELISA BrdU Colorimetric, Roche), and we have performed the assay 5 hours after BrdU addition. Experiments with MIN6 cells were repeated 3 times, each one using 10 replicates; those experiments performed with dispersed islets cells were repeated 3 times, each one using 6 replicates.

### Mouse model

Wild type (WT) and PTP1B deficient mice (PTP1B ^−^/^−^) were obtained from Abbott Laboratories [10], [18]. All experiments were performed in 8 weeks old male mice littermates, on a mixed C57BL/6Jx129 background. The animals were housed with a 12-h light/12-h dark cycle in temperature and humidity-controlled environment in animal facility with free access to water and standard laboratory chow.

### Islet studies

Animals were sacrificed by cervical dislocation after being fully anesthetized by ketamine-xylacine. After direct puncture of the common bile duct, a collagenase solution was perfused to digest the pancreas, and islets were purified using Histopaque gradient [19]. Islets were handpicked under a stereomicroscope and separate batches of 8 islets were used to determine insulin secretion and content in static incubation assays as previously described [20]. Insulin was measured by using a mouse insulin ELISA kit (Mercodia). We have performed four different experiments. In each one we have pooled islets from two WT or two PTP1B −/− mice, as we need at least 80 islets (similar in size) per genotype to incubate the different batches with low and high glucose concentrations.

### Pancreas morphometry and immunolocalization procedures

Pancreases were obtained, fixed overnight in 10% formalin and paraffin-embebed. Three non-consecutive 4-µm thick pancreatic sections 150 µm apart (six sections/animal) were labelled with a standard immunofluorescence method for paraffin sections. Primary antibodies used were: guinea pig anti-insulin IgG (1∶1000 dilution, Dako, Glostrup, Denmark) and rabbit anti-glucagon IgG (1∶500 dilution, Dako). Cy3 anti-guinea pig and Cy2 anti-rabbit labelled secondary antibodies (1∶500 dilution, Jackson Immunoresearch, Suffolk, UK) were used. Hoescht (1∶500 dilution, Sigma-Aldrich) was employed as nuclear marker. Images were taken with a Leyca DMR HC epifluorescence microscope. For morphometric analysis, at least 100 islets from both genotypes were manually traced and analyzed using Image J (National Institutes of Health, Bethesda, MD, USA; http://rsb.info.nih.gov/ij/) software. α and β-cell mass were quantified blindly as α and β-cell volume density respectively, multiplied by pancreas weight [21].

An immunofluorescence approach was employed in order to study subcellular localization of FOXO1 in islets. For this experiment, primary antibodies used were: guinea pig anti-insulin IgG (1∶1000 dilution, Dako) and rabbit anti-FOXO1 IgG (1∶20 dilution, Cell Signaling, Beverly, MA, USA). Cy3 anti-guinea pig and Cy2 anti-rabbit labelled secondary (1∶500 dilution, Jackson Immunoresearch) antibodies were used. Hoescht antibody (1∶500 dilution, Sigma-Aldrich) was used as nuclear marker. Images were taken as described above and analyzed using Image J software. The results are expressed as the number of β-cells co-expressing insulin and total/nuclear FOXO1.

### β-cell proliferation and apoptosis measurements

Three non-consecutive 4-µm sections were deparaffinised, rehydrated and treated with citrate buffer (10 mmol/l; pH 6.0) as antigen retrieval. Slides were immunostained with purified mouse anti-ki67 antibody (1∶50 dilution, BD Pharmigen, San Jose, CA, USA), and cleaved caspase-3 (1∶400 dilution, Cell Signaling) in order to study proliferation and apoptosis respectively. At least 3000 beta cells per sample were counted. Co-localization with insulin positive β-cells was checked individually. Image analysis was performed as stated above. The results are expressed as the number of β-cells co-expressing insulin and ki67 or cleaved caspase-3.

### Western Blot

Protein extracts from islets and MIN6 transfected cells were prepared in lysis buffer (50 mmol/l Tris pH 7.5, 5 mmol/l EDTA, 150 mmol/l NaCl, 1% Triton X-100, 10 mmol/l sodium phosphate) containing fresh protease and phosphatase inhibitor cocktails (Roche, Basel, Switzerland). Proteins were separated by 8% SDS-PAGE and transferred to nitrocellulose membranes. Immunoblots were performed using the following antibodies: rabbit monoclonal anti-PTP1B antibody (Novus Biologicals, Littleton, CO, USA), rabbit anti-STAT3 antibody, rabbit anti-pSTAT3^Tyr705^ antibody, rabbit anti-AKT IgG, rabbit anti-pAKT^Thr308^ antibody, rabbit anti-ERK1/2 (p44/42 MAPK) antibody, rabbit anti-pERK^Thr202/Tyr204^ antibody, rabbit anti-FOXO1 IgG, rabbit anti-p FOXO1^Ser256^ antibody (all of them from Cell Signaling) and mouse monoclonal anti-p53 antibody (ABCAM, Cambridge, UK). As a loading control, rabbit anti-actin antibody was used (Sigma Aldrich). The antibody dilution used was 1∶1000.

Immunoblots were developed with horseradish peroxidise-conjugated secondary antibodies (GE Healthcare Bio-Sciences Corp. Piscataway, NJ, USA) and visualized using enhanced chemiluminiscence (Thermo Fisher Scientific Inc. Waltham, MA, USA). Bands were detected by an ImageQuant LAS 4000 camera (GE Healthcare) and quantified by densitometry scanning with Image J software.

### Intraperitoneal glucose tolerance test

In order to assess glucose tolerance, intraperitoneal glucose tolerance tests (ipGTT) were performed after overnight fasting in PTP1B ^−^/^−^ and WT mice with free access to water. The ipGTT was performed by the administration of an injection of D-glucose (2 g/Kg body weight), and blood samples were collected from tail vein at 0, 15, 30, 60 and 120 minutes after injection. Glycaemia was measured at the same time points using a clinical glucometer and Accutrend Check test strips (Roche Diagnostics, Switzerland). Plasma was obtained by blood centrifugation and kept at −80°C for insulin determination using a mouse insulin ELISA kit (Mercodia, Uppsala, Sweden).

### Diabetes induction

Diabetes was induced in 4-6 hours fasted 8 weeks-old male mice by a single intraperitoneal injection of 125 mg/Kg streptozotocin (STZ, Sigma-Aldrich, St Louis, MO, USA) diluted in 0.9% NaCl with 100 mmol/l sodium citrate (pH 4.5). The animals were monitored daily for hyperglycaemia for 6 days, and those mice that had an average of plasma blood glucose levels above 250 mg/dl were considered for the experiment. After the application of the selection criteria, mice were monitored weekly for seven weeks, measuring blood glucose levels. At the time of sacrifice, 7 weeks after the streptozotocin injection, pancreas samples were excised and conserved for performing further morphometric and proliferative/apoptotic studies, as described above.

### Statistical analysis

All the results are expressed as mean±SEM. Differences between experimental groups were determined by Students t-test. p<0.05 was considered statistical significant.

## Results

### PTP1B modulates proliferation in pancreatic β-cells

**Figure 1:**
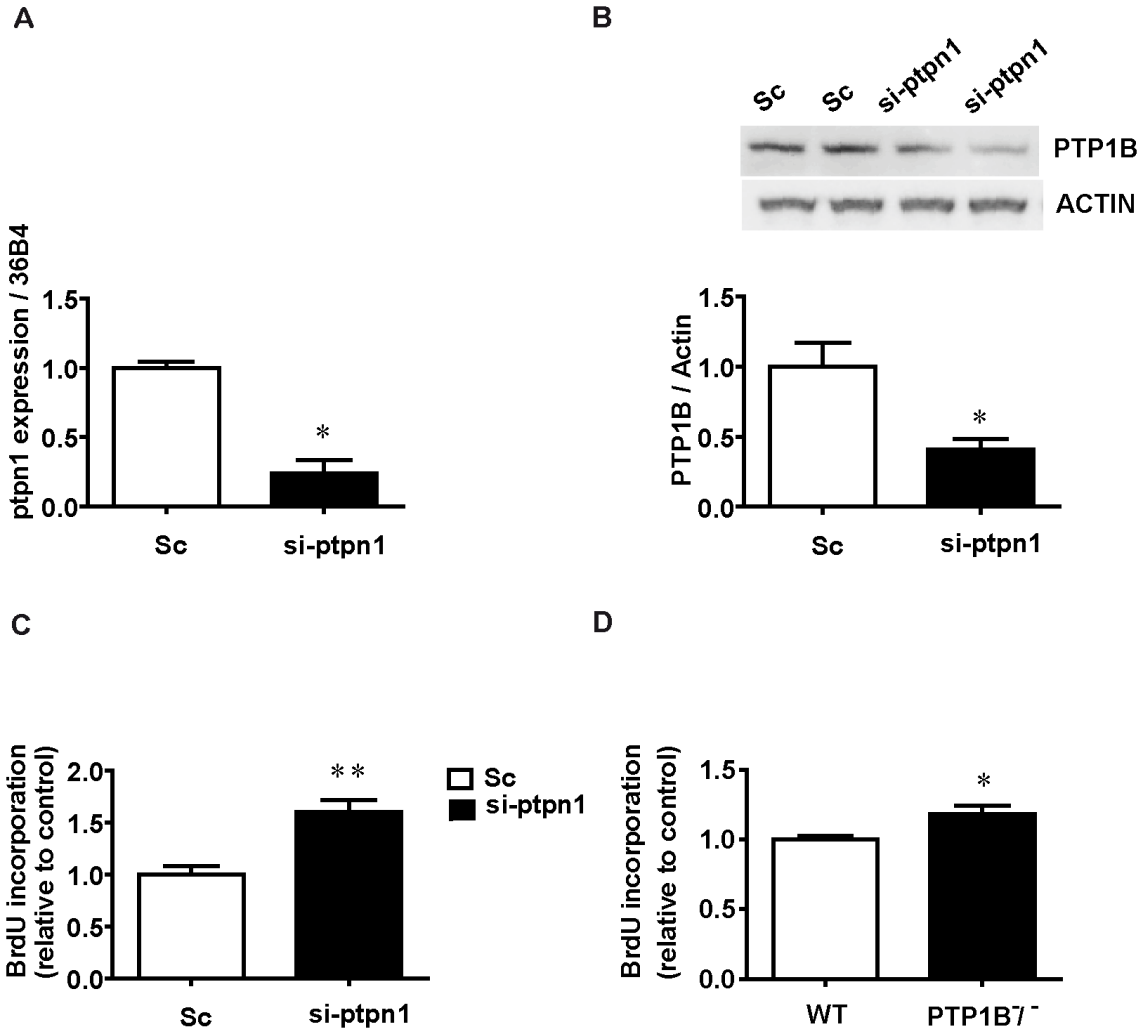
Ablation of PTP1B increases proliferation in *in vitro* transfected MIN6 cells and dispersed islet cells. A) mRNA expression and B) protein content are used to confirm the efficiency of PTP1B siRNA transfection and silencing in MIN6 cells. Values are expressed relative to 36B4 as a housekeeping gene and actin protein content respectively. C) *In vitro* BrdU incorporation in proliferating si-ptpn1 MIN6 cells. Represented values are normalized to scrambled siRNA (Sc) MIN6 cells; n = 3 different experiments, 10 replicates per group each experiment. D) *In vitro* BrdU incorporation in PTP1B ^−^/^−^ proliferating dispersed islet cells is expressed relative to WT; n = 3 different experiments, 6 replicates per group each experiment. All bars represent mean±SEM * p<0.05, ** p<0.005 PTP1B ^−^/^−^ *vs* WT.

In order to determine whether PTP1B regulates β-cell mass, we silenced this phosphatase in the mouse pancreatic β-cell line MIN-6 using siRNA (si-ptpn1). 100 nM of siRNA was effective in silencing PTP1B mRNA expression by 76% (Figure 1A) and reduce PTP1B protein content by 59% (Figure 1B) in MIN-6 cells compared with the scramble si-RNA (Sc). Our examination of si-ptpn1 MIN6 cells showed a significantly increased proliferative rate on those cells when assessed by measuring *in vitro* BrdU incorporation (Figure 1C). Interestingly, we have confirmed the role for PTP1B ^−^/^−^ in regulating islet cell proliferation in dispersed islets from PTP1B ^−^/^−^ and WT mice by performing the same experiments of *in vitro* incorporation of BrdU in this setting (Figure 1D).

### PTP1B regulates changes in islet morphometry

**Figure 2:**
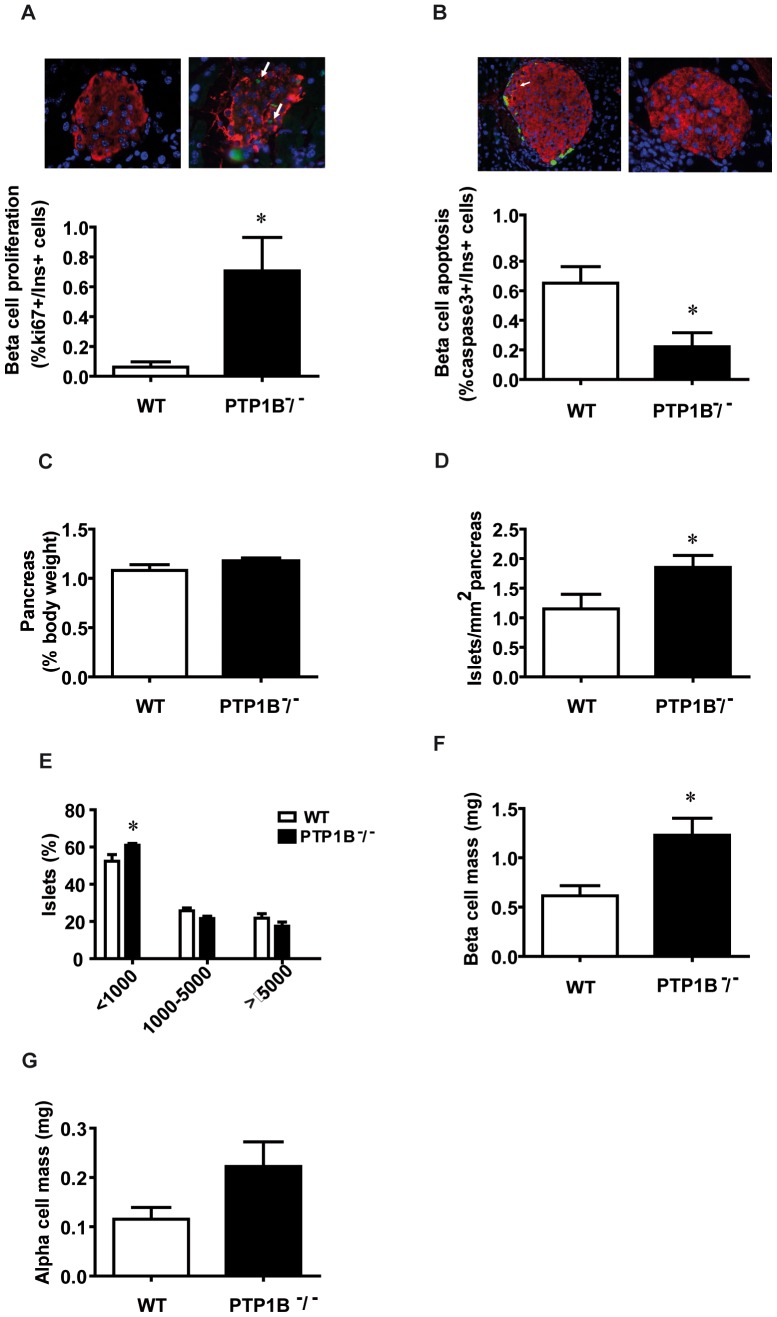
Ablation of PTP1B increases proliferation in *in vivo* β-cells and alters pancreas morphometry in PTP1B ^−^/^−^ mice. Morphometric analysis of fixed paraffin embedded pancreas from PTP1B ^−^/^−^ and WT mice (n = 5–7 animals per group). A) Level of proliferating β-cells (ki67+/insulin+) from PTP1B ^−^/^−^ and WT mice. Representative images showing immunostaining for ki67 (green), insulin (red), and merged images together with Dapi for nuclei (blue) on pancreatic sections from PTP1B ^−^/^−^ and WT mice. B) Levels of apoptotic β-cells (caspase3+/insulin+) from PTP1B ^−^/^−^ and WT mice. Representative images showing immunostaining for insulin (red), Caspase3 (green), and Dapi for nuclei (blue) on pancreatic sections from PTP1B ^−^/^−^ and WT mice. C) Pancreas weight normalized by body weight. D) Number of islets, over the total pancreatic area (µm^2^) studied. E) Distribution of islets on the basis of their size, expressed as the percentage of a given size, over the total pancreatic area (µm^2^) studied. F) β-cell mass is quantified blindly as β-cell volume density, multiplied by pancreas weight. G) α-cell mass is quantified blindly as α-cell volume density, multiplied by pancreas weight. All bars represent mean±SEM * p<0.05 PTP1B ^−^/^−^ *vs* WT.

As we have stated above, it seems clear that PTP1B plays a determinant role in pancreatic β-cell proliferation. Therefore, to confirm our findings we next performed *ex vivo* morphometric analysis of pancreases embedded in paraffin, both from PTP1B ^−^/^−^ and WT mice. On this regard, islets from PTP1B ^−^/^−^ mice also exhibited higher β-cell proliferation (Figure 2A), measured by ki67 co-expression with insulin. Interestingly, β-cell apoptosis was lower in islets from PTP1B ^−^/^−^ mice compared to WT islets (Figure 2B), measured by cleaved-caspase3 co-expression with insulin.

Proliferation and apoptosis are important molecular mechanisms determining cellular mass. Although no differences in pancreas weight were observed when normalized by body weight (Figure 2C), the morphometric analysis of the pancreas showed a higher number of islets in PTP1B ^−^/^−^ versus WT littermate mice (Figure 2D). A more detailed study of islet size showed that the increase in islet area from PTP1B ^−^/^−^ mice is mainly due to a higher number of small islets (<1000 um^2^) (Figure 2E). We have also observed a significantly increase in β-cell mass in PTP1B ^−^/^−^ when compared with WT mice (Figure 2F). The α-cell mass showed a tendency to be increased in PTP1B ^−^/^−^ mice, although this difference didn’t achieve statistical significance (Figure 2G).

### PTP1B modulates signalling pathways involved in the regulation of beta cell mass in isolated pancreatic islets

**Figure 3:**
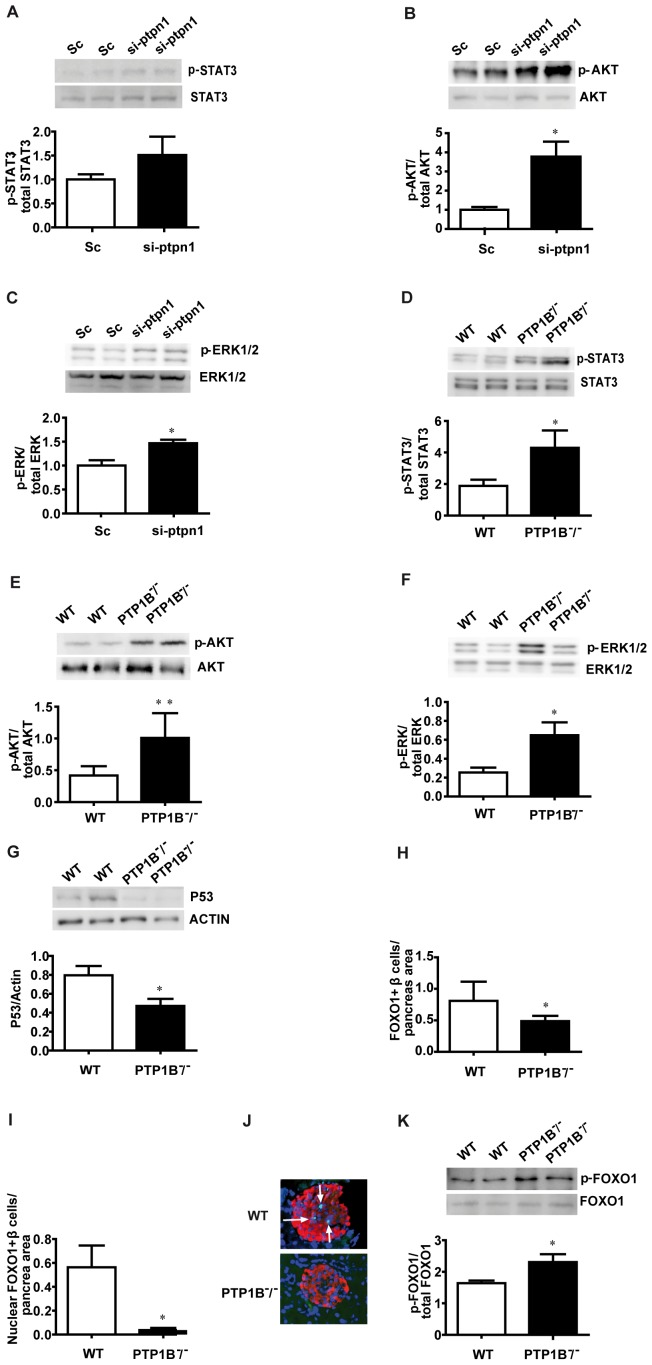
Pro-proliferative/pro-survival signalling in in vitro si-ptpn1 MIN6 cells and in isolated pancreatic islets from 8 weeks old PTP1B ^−^/^−^ mice. A-C) Western blot of phospho-STAT3^Tyr705^, phospho-AKT^Thr308^ and phospho-ERK1/2^Thr202^/^Tyr204^ in transfected MIN6 cells. Representative immunoblot for each phosphorylated and total protein is shown. n = 3 per group. Bands are quantified by densitometry and values expressed as the ratio of each phosphorylated form relative to the total protein expression. D-G) Representative immunoblots and quantification for phospho-STAT3^Tyr705^, phospho-AKT^Thr308^, phospho-ERK1/2^Thr202^/^Tyr204^ and p53 in isolated islets from PTP1B ^−^/^−^ and WT mice. Bands are quantified by densitometry and values expressed as the ratio of each phosphorylated form relative to the total protein expression (immunoblots for total STAT3, AKT and ERK1/2 are also shown, together with actin in the case of p53). H) Levels of FOXO1 protein expression in β-cell, determined by immunofluorescence analysis. I) FOXO1 nuclear localization in islets is represented over the total pancreatic area (µm^2^) studied. J) Representative images of FOXO1 in WT and PTP1B ^−^/^−^. K) Quantification and representative immunoblot for phospho-FOXO1^Ser256^ in isolated islets from PTP1B ^−^/^−^ and WT mice. n = 6 animals per group. All bars represent mean±SEM * p<0.05, ** p<0.005 si-ptpn1 *vs* Sc or PTP1B ^−^/^−^ *vs* WT.

After establishing a role for PTP1B in the regulation of β-cell proliferation, we further assess the potential molecular mechanisms responsible for these changes in MIN6 cells and islets from PTP1B ^−^/^−^ and WT mice. As PTP1B is a main negative regulator of insulin signalling, it would be feasible to think that its ablation could affect key proteins of the different pathways regulated by insulin involved in β-cell proliferation and apoptosis [7], [22], [23]. On this regard, we found that AKT and ERK1/2 phosphorylation were significantly increased in si-ptpn1 MIN6 cells (Figure 3B and 3C). Similar results were found when we analyzed STAT3, AKT and ERK1/2 phosphorylation in pancreatic islets; all of them showed the same pattern described in MIN6 cells as its phosphorylation status is significantly increased in islets from PTP1B ^−^/^−^ mice when compared to their WT littermates (Figure 3D-F). On the other hand, we assessed the expression of the pro-apoptotic protein p53 which was significantly decreased in islets from PTP1B ^−^/^−^ mice (Figure 3G), which supports the role played by PTP1B not only in the control of cell proliferation but also in regulating cell apoptosis (Figure 2A and 2B).

Likewise, we have studied the insulin stimulated AKT/FOXO1 signalling, which could be involved in regulating proliferation and/or apoptosis in β-cells downstream of PTP1B [16], [24]. Immunohistochemical analysis of pancreatic islets showed that FOXO1 positive β-cells were significantly reduced in PTP1B ^−^/^−^ mice when compared with their respective WT littermates (Figure 3H). Moreover, regulation of the nuclear/cytoplasmic distribution of FOXO1 is important in determining its biological activity in metabolism, cellular proliferation and survival rather than protein abundance *per se*. We found that FOXO1 nuclear localization was reduced in PTP1B ^−^/^−^ islets (Figure 3I and 3J), concomitantly with an increased FOXO1 phosphorylation at Ser^256^ (Figure 3K).

### PTP1B regulates β-cell insulin secretion in isolated islets and improves glucose tolerance and insulin response *in vivo*

**Figure 4:**
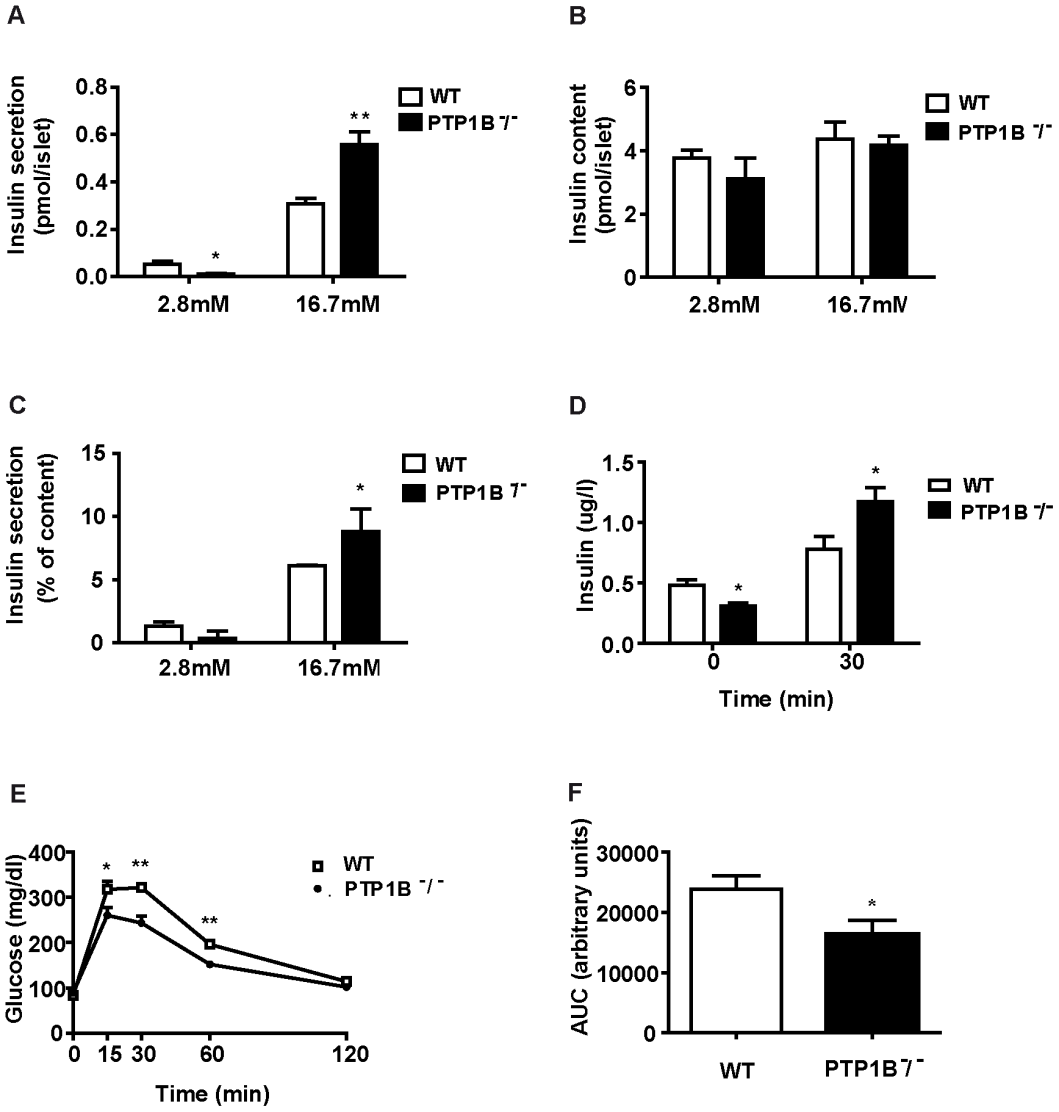
Glucose induced insulin secretion both *in vivo* and in isolated islets from PTP1B ^−^/^−^ and WT mice. Islets were isolated from 8 weeks old WT and PTP1B ^−^/^−^ mice and insulin secretion was assayed at indicated glucose concentrations (2.8 and 16.7 mM) in static incubation experiments as indicated in materials and methods section. A) Insulin secretion per islet. B) Islet insulin content. C) Insulin secretion as a percentage of total insulin content. D) Plasma insulin levels after glucose administration during an ipGTT test in PTP1B ^−^/^−^ (n = 5) and WT (n = 5) mice. E) Glucose tolerance test (ipGTT): blood glucose levels of PTP1B ^−^/^−^ (n = 13) and WT (n = 12) mice at the indicated time points after an intraperitoneal injection of glucose (2 g/Kg body weight). F) ipGTT area under the curve (AUC). AUC was calculated using the trapezoidal rule. All results represent mean±SEM * p<0.05; ** p<0.005 PTP1B ^−^/^−^ *vs* WT.

Our next aim was to see whether PTP1B actions on β-cell have an effect on insulin secretion; for such purpose we undertook studies both *in vitro* and *in vivo*. *In vitro* basal insulin secretion (2.8 mM glucose) was lower in PTP1B ^−^/^−^ than in WT islets (Figure 4A), although after normalizing by insulin content, which was similar between PTP1B ^−^/^−^ and WT pancreatic islets (Figure 4B), insulin secretion does not achieve statistical significance (Figure 4C) (p = 0.06). When islets were stimulated with high glucose concentrations (16.7 mM glucose) PTP1B ^−^/^−^ islets secreted significantly more insulin than the islets from their WT littermates (Figure 4A and 4C). These *in vitro* observations suggest that PTP1B could directly modulate insulin secretion, although paracrine effects from other cell types within the islet cańt be ruled out. Our *in vivo* experiments showed that PTP1B ^−^/^−^ mice were hypoinsulinemic after an overnight fasting and that at 30 minutes during ipGTT plasma insulin levels were significantly higher in PTP1B ^−^/^−^ mice (Figure 4D), in line with our *in vitro* studies. This is consistent with the higher glucose tolerance observed in PTP1B ^−^/^−^ mice, as reflected by differences in glycaemia during an intraperitoneal glucose tolerance test (ipGTT) (Figures 4E, F). These differences in glucose handling are in agreement with previous observations performed in peripheral tissues [9], [10]. In summary, our *in vitro* and *in vivo* islet results led us to conclude that the absence of PTP1B alters pancreatic β-cell performance by increasing insulin secretion under glucose stimulated conditions.

### PTP1B ablation alleviates streptozotocin-induced β-cell damage

**Figure 5:**
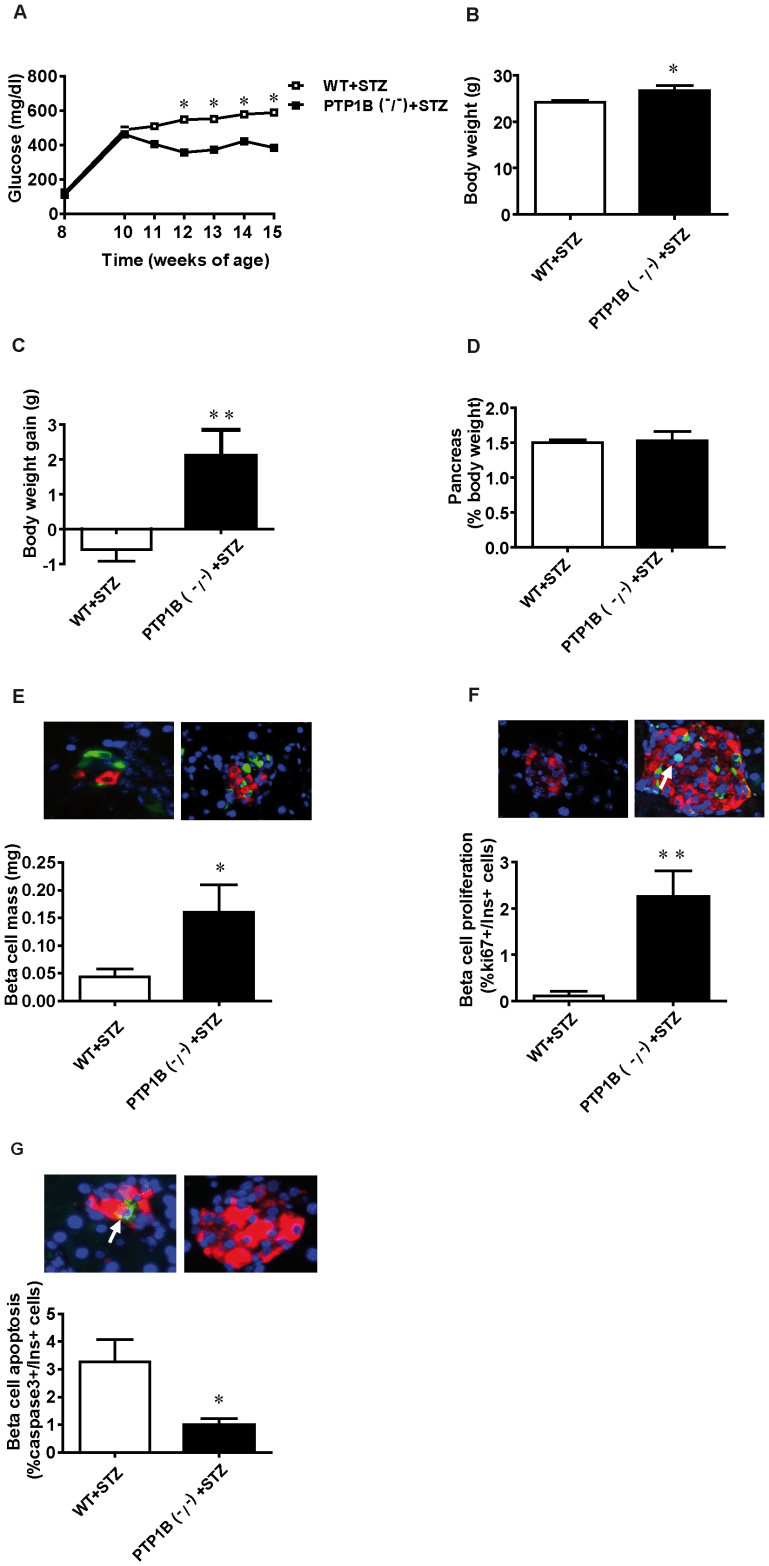
β-cell mass recovery after streptozotocin-induced diabetes in PTP1B ^−^/^−^ mice. A) Blood glucose levels along the experimental period (7 weeks) after STZ-induced β-cell damage in PTP1B ^−^/^−^ and WT fed mice. B) Body weight at the end of the experimental period after STZ-induced β-cell damage in PTP1B ^−^/^−^ and WT fed mice. C) Body weight gain 7 weeks after STZ injection in both experimental groups. D) Pancreas weight (normalized by body weight) of STZ-treated PTP1B ^−^/^−^ and WT fed mice 7 weeks after STZ injection. E) β-cell mass is quantified blindly as β-cell volume density, multiplied by pancreas weight (n = 6 animals per group). Representative images showing immunostaining for insulin (red), glucagon (green), and Dapi for nuclei (blue) on pancreatic sections from STZ-treated PTP1B ^−^/^−^ and WT mice. F) Levels of proliferating β-cells (ki67+/insulin+) in STZ-treated PTP1B ^−^/^−^ and WT mice (n = 6 animals per group). Representative images showing immunostaining for insulin (red), ki67 (green), and Dapi for nuclei (blue) on pancreatic sections from STZ-treated PTP1B ^−^/^−^ and WT mice. G) Levels of apoptotic β-cells (caspase3+/insulin+) in STZ-treated PTP1B ^−^/^−^ and WT mice. (n = 6 animals per group). Representative images showing immunostaining for insulin (red), Caspase3 (green), and Dapi for nuclei (blue) on pancreatic sections from STZ-treated PTP1B ^−^/^−^ and WT mice. All results represent mean±SEM; * p<0.05, ** p<0.005 PTP1B ^−^/^−^ + STZ *vs* WT + STZ.

Administration of streptozotocin (STZ) to mice causes damage and loss of β-cells leading to a chronic hyperglycaemic state. This model has been used as a model of β-cell regeneration as partial recovery of β-cell mass can be reached under certain conditions [25]. Given our previous results demonstrating an effect of PTP1B on β-cell proliferation and apoptosis, we tested whether the absence of PTP1B could potentiate β-cell mass recovery. STZ treatment was effective in inducing diabetes both in PTP1B ^−^/^−^ and WT mice, based on the high blood glucose levels observed during the first 6 days of treatment (above 250 mg/dl, inclusion criteria). Hyperglycaemia was significantly lower in STZ-treated PTP1B ^−^/^−^ mice than in STZ-treated WT littermates (Figure 5A) along the 7 weeks of the experimental period. The absence of PTP1B also ameliorates the decrease in body weight caused by the diabetic state (Figure 5B and C), measured at the end of the procedure, when mice were 15 weeks of age. Pancreas weight shows no differences between STZ-treated PTP1B ^−^/^−^ and WT mice (Figure 5D).

To study whether the improvement in glycaemia observed in STZ-treated PTP1B ^−^/^−^ mice was due to a specific effect in the pancreas, we performed morphometric studies in fixed pancreas from STZ-treated WT and PTP1B ^−^/^−^ mice 7 weeks after STZ injection. β-cell mass was significantly higher in STZ-treated PTP1B ^−^/^−^ mice (Figure 5E) when compared with islets from STZ-treated WT littermates. This increase in β-cell mass is consistent with the observed increase in β-cell proliferation (Figure 5F) in STZ-treated PTP1B ^−^/^−^ mice. Moreover, β-cell apoptosis was decreased in islets from STZ-treated PTP1B ^−^/^−^ mice when compared with STZ-treated WT littermates (Figure 5G). Thus, we have also demonstrated that deletion of PTP1B could improve β-cell mass and survival in an animal model of diabetes.

## Discussion

PTP1B plays a major role in several physiological functions including energy balance and the regulation of glucose homeostasis [26]–[28], hence it is a potential target for the treatment of Diabetes. On this regard, much effort has been done in order to describe the role of PTP1B in peripheral tissues [11]–[14], [29], but information about its role in endocrine pancreas is limited [15], [16]. To our knowledge, this is the first study showing that PTP1B modulates β-cell mass in a cell autonomous manner through the regulation of key signalling pathways involved in β cell proliferation and apoptosis.

This work shows a higher ERK1/2 and AKT phosphorylation in the β-cell line MIN6 once PTP1B is silenced, as well as in isolated islets from PTP1B ^−^/^−^ mice. Once ERK1/2 is activated it translocates to the nucleus, where through interaction with transcription factors regulates cell proliferation and survival [30]. Activation of AKT by insulin signalling has also been linked to β-cell survival [31], [32] and to the degradation by the proteasome of the pro-apoptotic protein p53 [33]. A plausible explanation for the mitogenic effect of AKT activation is that this kinase phosphorylates and inactivates the transcription factor forkhead box O1 (FOXO1), which negatively regulates β-cell formation and function. FOXO1 transcriptional activity is inhibited via nuclear exclusion [34]–[39], and its phosphorylation is believed to be one of the main anti-apoptotic signals downstream of AKT [40]. Although its role in β-cell is currently under debate [41], our results are in agreement with those of Folli *et al.* [39], as we here show that FOXO1 nuclear localization is reduced in β-cells from PTP1B ^−^/^−^ mice concomitantly with an increased phosphorylation of FOXO1 at residue Ser^256^ in islets from PTP1B ^−^/^−^ mice [42]. Folli *et al*. [39] showed that when disrupting insulin signalling in β-cell (βIRKO mice), FOXO1 locates mainly in the nucleus, avoiding cell cycle progression and leading to a reduction in β-cell mass. Our novel data about the role played by PTP1B in β-cell proliferation are in agreement with previous observations in hepatocytes showing that this phosphatase regulates multiple signalling pathways that trigger proliferation in response to a partial hepatectomy [43].

Our results showed that the increase in AKT phosphorylation is in line with a decrease in the expression of p53 protein in PTP1B ^−^/^−^ islets. In addition, p53 expression is regulated via the JAK2/STAT3 signalling pathway, which is in turn negatively regulated by PTP1B [44]. Thus, it has been shown that the dephosphorylation of p-STAT3 by PTP1B lead to the stabilization of p53 expression and apoptosis [45]. We here suggest that the combination of AKT activation and the increased phosphorylation of STAT3, facilitated by the lack of PTP1B, allows a decrease in p53 protein level, which partially explain the lower beta cell apoptosis described in PTP1B ^−^/^−^ islets. These results are in accordance with the higher β-cell proliferation observed both in si-ptpn1 MIN6 cells and isolated islets from PTP1B ^−^/^−^ mice.

Our studies regarding *in vivo* glucose homeostasis in 8 weeks-old PTP1B ^−^/^−^ male mice unravelled an enhanced insulin secretion during the ipGTT. These results correlate with the higher glucose-stimulated insulin release observed in isolated pancreatic islets from those mice, and are in agreement with a previous report performed in rat islets, in which *in vitro* silencing of PTP1B leads to an increase in insulin release [46]. Moreover, pancreas morphometric analyses have revealed a higher β-cell mass in PTP1B ^−^/^−^ than in WT mice. A detailed study of these islets led us to conclude that the increase in β-cell mass is due to a significantly higher number of the smallest islets (under 1000µm^2^) in PTP1B ^−^/^−^ than in WT mice. Together, these results confirm that the effect of PTP1B in β-cell is cell autonomous, and independent of the higher whole body insulin sensitivity observed in PTP1B ^−^/^−^ mice at 8 weeks of age. However, unpublished results in our laboratory regarding glucose homeostasis and pancreas morphometry in PTP1B ^−^/^−^ mice at 15 weeks are in agreement with previous studies [15], [16] which have evaluated β-cell morphometry in PTP1B ^−^/^−^ mice showing no differences between pancreatic β-cell cross-sectional area of 12 and 15 weeks old PTP1B ^−^/^−^ and WT mice. These results seem to indicate that the higher peripheral insulin sensitivity observed in PTP1B ^−^/^−^ mice is not able to counteract the effect of the absence of PTP1B on β-cell physiology until later ages.

To investigate the potential role of PTP1B in the mechanisms involved in the characteristic β-cell loss associated with the progression of diabetes, we used the streptozotocin-induced pancreatic injury model, as a way to evaluate the effect of a stable hyperglycaemic state. Plasma glucose levels are significantly lower in PTP1B ^−^/^−^ mice after streptozotocin-induced diabetes. This moderate improvement in glucose handling could be explained by a higher β-cell mass in PTP1B ^–^/^–^ mice due to a higher rate of β-cell replication, measured at the end of the experimental period, 7 weeks after the streptozotocin injection. In addition to this, PTP1B ablation may be involved in protecting β-cells from glucotoxicity-induced apoptotic cell death, as shown by our caspase3 immunostaining analysis. These results are in line with a previous report where deletion of PTP1B is able to partially recover β-cell damage induced by the genetic ablation of IRS2, a model of genetically induced Type 2 Diabetes [15].

In summary, our results support the notion that PTP1B is a critical regulator of β-cell physiology both at the level of β-cell mass and function. The information presented in this manuscript led us to propose PTP1B as a potential target for the treatment of β-cell dysfunction, critical in Type 2 Diabetes aetiology. Moreover, our data underscore the importance of future studies aimed to further delineate PTP1B actions in pancreatic β-cell.

## References

[1] Lebovitz HE (2001) Insulin resistance: definition and consequences. Exp Clin Endocrinol Diabetes 109 Suppl 2S135–148. 11460565 10.1055/s-2001-18576

[2] Benito M (2011) Tissue-specificity of insulin action and resistance. Arch Physiol Biochem 117: 96–104. 21506723 10.3109/13813455.2011.563748

[3] Henquin JC, Cerasi E, Efendic S, Steiner DF, Boitard C (2008) Pancreatic beta-cell mass or beta-cell function? That is the question! Diabetes Obes Metab 10 Suppl 41–4. 18834427 10.1111/j.1463-1326.2008.00968.x

[4] Butler AE, Janson J, Bonner-Weir S, Ritzel R, Rizza RA, et al (2003) Beta-cell deficit and increased beta-cell apoptosis in humans with type 2 diabetes. Diabetes 52: 102–110. 12502499 10.2337/diabetes.52.1.102

[5] Otani K, Kulkarni RN, Baldwin AC, Krutzfeldt J, Ueki K, et al (2004) Reduced beta-cell mass and altered glucose sensing impair insulin-secretory function in betaIRKO mice. Am J Physiol Endocrinol Metab 286: E41–49. 14519599 10.1152/ajpendo.00533.2001

[6] Ueki K, Okada T, Hu J, Liew CW, Assmann A, et al (2006) Total insulin and IGF-I resistance in pancreatic beta cells causes overt diabetes. Nat Genet 38: 583–588. 16642022 10.1038/ng1787

[7] Taniguchi CM, Emanuelli B, Kahn CR (2006) Critical nodes in signalling pathways: insights into insulin action. Nat Rev Mol Cell Biol 7: 85–96. 16493415 10.1038/nrm1837

[8] Xue B, Kim YB, Lee A, Toschi E, Bonner-Weir S, et al (2007) Protein-tyrosine phosphatase 1B deficiency reduces insulin resistance and the diabetic phenotype in mice with polygenic insulin resistance. J Biol Chem 282: 23829–23840. 17545163 10.1074/jbc.M609680200

[9] Elchebly M, Payette P, Michaliszyn E, Cromlish W, Collins S, et al (1999) Increased insulin sensitivity and obesity resistance in mice lacking the protein tyrosine phosphatase-1B gene. Science 283: 1544–1548. 10066179 10.1126/science.283.5407.1544

[10] Klaman LD, Boss O, Peroni OD, Kim JK, Martino JL, et al (2000) Increased energy expenditure, decreased adiposity, and tissue-specific insulin sensitivity in protein-tyrosine phosphatase 1B-deficient mice. Mol Cell Biol 20: 5479–5489. 10891488 10.1128/mcb.20.15.5479-5489.2000 PMC85999

[11] Bence KK, Delibegovic M, Xue B, Gorgun CZ, Hotamisligil GS, et al (2006) Neuronal PTP1B regulates body weight, adiposity and leptin action. Nat Med 12: 917–924. 16845389 10.1038/nm1435

[12] Owen C, Czopek A, Agouni A, Grant L, Judson R, et al (2012) Adipocyte-specific protein tyrosine phosphatase 1B deletion increases lipogenesis, adipocyte cell size and is a minor regulator of glucose homeostasis. PLoS One 7: e32700. 22389718 10.1371/journal.pone.0032700 PMC3289674

[13] Delibegovic M, Zimmer D, Kauffman C, Rak K, Hong EG, et al (2009) Liver-specific deletion of protein-tyrosine phosphatase 1B (PTP1B) improves metabolic syndrome and attenuates diet-induced endoplasmic reticulum stress. Diabetes 58: 590–599. 19074988 10.2337/db08-0913 PMC2646057

[14] Delibegovic M, Bence KK, Mody N, Hong EG, Ko HJ, et al (2007) Improved glucose homeostasis in mice with muscle-specific deletion of protein-tyrosine phosphatase 1B. Mol Cell Biol 27: 7727–7734. 17724080 10.1128/MCB.00959-07 PMC2169063

[15] Kushner JA, Haj FG, Klaman LD, Dow MA, Kahn BB, et al (2004) Islet-sparing effects of protein tyrosine phosphatase-1b deficiency delays onset of diabetes in IRS2 knockout mice. Diabetes 53: 61–66. 14693698 10.2337/diabetes.53.1.61

[16] Gonzalez-Rodriguez A, Mas-Gutierrez JA, Mirasierra M, Fernandez-Perez A, Lee YJ, et al (2012) Essential role of protein tyrosine phosphatase 1B in obesity-induced inflammation and peripheral insulin resistance during aging. Aging Cell 11: 284–296. 22221695 10.1111/j.1474-9726.2011.00786.x PMC3306541

[17] Miyazaki J, Araki K, Yamato E, Ikegami H, Asano T, et al (1990) Establishment of a pancreatic beta cell line that retains glucose-inducible insulin secretion: special reference to expression of glucose transporter isoforms. Endocrinology 127: 126–132. 2163307 10.1210/endo-127-1-126

[18] Gonzalez-Rodriguez A, Mas Gutierrez JA, Sanz-Gonzalez S, Ros M, Burks DJ, et al (2010) Inhibition of PTP1B restores IRS1-mediated hepatic insulin signaling in IRS2-deficient mice. Diabetes 59: 588–599. 20028942 10.2337/db09-0796 PMC2828646

[19] Casas S, Novials A, Reimann F, Gomis R, Gribble FM (2008) Calcium elevation in mouse pancreatic beta cells evoked by extracellular human islet amyloid polypeptide involves activation of the mechanosensitive ion channel TRPV4. Diabetologia 51: 2252–2262. 18751967 10.1007/s00125-008-1111-z PMC7212067

[20] Altirriba J, Gasa R, Casas S, Ramirez-Bajo MJ, Ros S, et al (2010) The role of transmembrane protein 27 (TMEM27) in islet physiology and its potential use as a beta cell mass biomarker. Diabetologia 53: 1406–1414. 20386877 10.1007/s00125-010-1728-6 PMC7096040

[21] Bonner-Weir S (2001) beta-cell turnover: its assessment and implications. Diabetes 50 Suppl 1S20–24. 11272192 10.2337/diabetes.50.2007.s20

[22] Avruch J (1998) Insulin signal transduction through protein kinase cascades. Mol Cell Biochem 182: 31–48. 9609112

[23] Mashili F, Chibalin AV, Krook A, Zierath JR (2013) Constitutive STAT3 phosphorylation contributes to skeletal muscle insulin resistance in type 2 diabetes. Diabetes 62: 457–465. 23043161 10.2337/db12-0337 PMC3554355

[24] Gonzalez-Rodriguez A, Escribano O, Alba J, Rondinone CM, Benito M, et al (2007) Levels of protein tyrosine phosphatase 1B determine susceptibility to apoptosis in serum-deprived hepatocytes. J Cell Physiol 212: 76–88. 17323378 10.1002/jcp.21004

[25] Zhang J, Zhang N, Liu M, Li X, Zhou L, et al (2012) Disruption of growth factor receptor-binding protein 10 in the pancreas enhances beta-cell proliferation and protects mice from streptozotocin-induced beta-cell apoptosis. Diabetes 61: 3189–3198. 22923474 10.2337/db12-0249 PMC3501856

[26] Popov D (2011) Novel protein tyrosine phosphatase 1B inhibitors: interaction requirements for improved intracellular efficacy in type 2 diabetes mellitus and obesity control. Biochem Biophys Res Commun 410: 377–381. 21683066 10.1016/j.bbrc.2011.06.009

[27] Tsou RC, Bence KK (2012) Central regulation of metabolism by protein tyrosine phosphatases. Front Neurosci 6: 192. 23308070 10.3389/fnins.2012.00192 PMC3538333

[28] Banno R, Zimmer D, De Jonghe BC, Atienza M, Rak K, et al (2010) PTP1B and SHP2 in POMC neurons reciprocally regulate energy balance in mice. J Clin Invest 120: 720–734. 20160350 10.1172/JCI39620 PMC2827947

[29] Bettaieb A, Matsuo K, Matsuo I, Wang S, Melhem R, et al (2012) Protein tyrosine phosphatase 1B deficiency potentiates PERK/eIF2alpha signaling in brown adipocytes. PLoS One 7: e34412. 22509299 10.1371/journal.pone.0034412 PMC3317973

[30] Mebratu Y, Tesfaigzi Y (2009) How ERK1/2 activation controls cell proliferation and cell death: Is subcellular localization the answer? Cell Cycle 8: 1168–1175. 19282669 10.4161/cc.8.8.8147 PMC2728430

[31] Wrede CE, Dickson LM, Lingohr MK, Briaud I, Rhodes CJ (2002) Protein kinase B/Akt prevents fatty acid-induced apoptosis in pancreatic beta-cells (INS-1). J Biol Chem 277: 49676–49684. 12393870 10.1074/jbc.M208756200

[32] Tuttle RL, Gill NS, Pugh W, Lee JP, Koeberlein B, et al (2001) Regulation of pancreatic beta-cell growth and survival by the serine/threonine protein kinase Akt1/PKBalpha. Nat Med 7: 1133–1137. 11590437 10.1038/nm1001-1133

[33] Ogawara Y, Kishishita S, Obata T, Isazawa Y, Suzuki T, et al (2002) Akt enhances Mdm2-mediated ubiquitination and degradation of p53. J Biol Chem 277: 21843–21850. 11923280 10.1074/jbc.M109745200

[34] Kousteni S (2012) FoxO1, the transcriptional chief of staff of energy metabolism. Bone 50: 437–443. 21816244 10.1016/j.bone.2011.06.034 PMC3228887

[35] Brunet A, Bonni A, Zigmond MJ, Lin MZ, Juo P, et al (1999) Akt promotes cell survival by phosphorylating and inhibiting a Forkhead transcription factor. Cell 96: 857–868. 10102273 10.1016/s0092-8674(00)80595-4

[36] Kops GJ, de Ruiter ND, De Vries-Smits AM, Powell DR, Bos JL, et al (1999) Direct control of the Forkhead transcription factor AFX by protein kinase B. Nature. 398: 630–634. 10.1038/19328 10217147

[37] Guo S, Rena G, Cichy S, He X, Cohen P, et al (1999) Phosphorylation of serine 256 by protein kinase B disrupts transactivation by FKHR and mediates effects of insulin on insulin-like growth factor-binding protein-1 promoter activity through a conserved insulin response sequence. J Biol Chem 274: 17184–17192. 10358076 10.1074/jbc.274.24.17184

[38] Rena G, Prescott AR, Guo S, Cohen P, Unterman TG (2001) Roles of the forkhead in rhabdomyosarcoma (FKHR) phosphorylation sites in regulating 14-3-3 binding, transactivation and nuclear targetting. Biochem J 354: 605–612. 11237865 10.1042/0264-6021:3540605 PMC1221692

[39] Folli F, Okada T, Perego C, Gunton J, Liew CW, et al (2011) Altered insulin receptor signalling and beta-cell cycle dynamics in type 2 diabetes mellitus. PLoS One 6: e28050. 22140505 10.1371/journal.pone.0028050 PMC3227614

[40] Alikhani M, Alikhani Z, Graves DT (2005) FOXO1 functions as a master switch that regulates gene expression necessary for tumor necrosis factor-induced fibroblast apoptosis. J Biol Chem 280: 12096–12102. 15632117 10.1074/jbc.M412171200

[41] Kobayashi M, Kikuchi O, Sasaki T, Kim HJ, Yokota-Hashimoto H, et al (2012) FoxO1 as a double-edged sword in the pancreas: analysis of pancreas- and beta-cell-specific FoxO1 knockout mice. Am J Physiol Endocrinol Metab 302: E603–613. 22215655 10.1152/ajpendo.00469.2011

[42] Zhang X, Gan L, Pan H, Guo S, He X, et al (2002) Phosphorylation of serine 256 suppresses transactivation by FKHR (FOXO1) by multiple mechanisms. Direct and indirect effects on nuclear/cytoplasmic shuttling and DNA binding. J Biol Chem 277: 45276–45284. 12228231 10.1074/jbc.M208063200

[43] Revuelta-Cervantes J, Mayoral R, Miranda S, Gonzalez-Rodriguez A, Fernandez M, et al (2011) Protein Tyrosine Phosphatase 1B (PTP1B) deficiency accelerates hepatic regeneration in mice. Am J Pathol 178: 1591–1604. 21406170 10.1016/j.ajpath.2010.12.020 PMC3078461

[44] Belin de Chantemele EJ, Muta K, Mintz J, Tremblay ML, Marrero MB, et al (2009) Protein tyrosine phosphatase 1B, a major regulator of leptin-mediated control of cardiovascular function. Circulation 120: 753–763. 19687357 10.1161/CIRCULATIONAHA.109.853077 PMC2736363

[45] Sainz-Perez A, Gary-Gouy H, Gaudin F, Maarof G, Marfaing-Koka A, et al (2008) IL-24 induces apoptosis of chronic lymphocytic leukemia B cells engaged into the cell cycle through dephosphorylation of STAT3 and stabilization of p53 expression. J Immunol 181: 6051–6060. 18941194 10.4049/jimmunol.181.9.6051

[46] Lu B, Wu H, Gu P, Du H, Shao J, et al (2012) Improved glucose-stimulated insulin secretion by intra-islet inhibition of protein-tyrosine phosphatase 1B expression in rats fed a high-fat diet. J Endocrinol Invest 35: 63–70. 21646858 10.3275/7766

